# Spoiling for a Fight: B Lymphocytes As Initiator and Effector Populations within Tertiary Lymphoid Organs in Autoimmunity and Transplantation

**DOI:** 10.3389/fimmu.2017.01639

**Published:** 2017-11-23

**Authors:** Jawaher Alsughayyir, Gavin J. Pettigrew, Reza Motallebzadeh

**Affiliations:** ^1^School of Clinical Medicine, University of Cambridge, Cambridge, United Kingdom; ^2^Division of Surgery and Interventional Science, University College London, London, United Kingdom; ^3^Institute of Immunity and Transplantation, University College London, London, United Kingdom; ^4^Department of Nephrology, Urology and Transplantation, Royal Free Hospital, London, United Kingdom

**Keywords:** B cells, tertiary lymphoid organs, autoimmunity, transplantation, germinal center

## Abstract

Tertiary lymphoid organs (TLOs) develop at ectopic sites within chronically inflamed tissues, such as in autoimmunity and rejecting organ allografts. TLOs differ structurally from canonical secondary lymphoid organs (SLOs), in that they lack a mantle zone and are not encapsulated, suggesting that they may provide unique immune function. A notable feature of TLOs is the frequent presence of structures typical of germinal centers (GCs). However, little is known about the role of such GCs, and in particular, it is not clear if the B cell response within is autonomous, or whether it synergizes with concurrent responses in SLOs. This review will discuss ectopic lymphoneogenesis and the role of the B cell in TLO formation and subsequent effector output in the context of autoimmunity and transplantation, with particular focus on the contribution of ectopic GCs to affinity maturation in humoral immune responses and to the potential breakdown of self-tolerance and development of humoral autoimmunity.

## Introduction

Adaptive immune responses are generally initiated within canonical secondary lymphoid organs (SLOs), such as lymph nodes (LN) and the spleen. SLOs are specially organized to facilitate presentation of antigen to the very small number of responder clones within the total lymphocyte population (1). Over the last few years, it has become increasingly evident that tertiary lymphoid organs (TLOs), also known as ectopic lymphoid tissue, can develop within peripheral organs in response to alloimmunity, chronic inflammation, cancer, chronic infection, and autoimmunity (2–6). Although TLOs have been described that contain predominantly a T cell infiltrate, a B cell component is generally also present, and often appears to dominate. This raises two fundamental questions: what role do B cells play in the genesis of TLOs; and what is their effector function once that TLO is established? The latter is particularly interesting, because although immunohistological evidence of complex germinal center (GC) activity is often detectable, a number of recent, seminal papers have reinforced the complexities of the GC reaction. The GC reaction is geared to producing high-affinity long-lived plasma cells (LLPCs) and memory B cells, but this requires precise spatiotemporal control of the key cellular interactions within the follicle. Here, we consider the role of the B cell, not only as a potential initiator in the formation of TLOs (in the context of solid-organ transplantation and autoimmunity) but also as a critical determinant of its output.

## Induction and Formation of TLOs—The Players

### The Initiators—B Cells or Not B Cells?

The initiation of lymphoid organogenesis requires the presence of hematopoietic CD4^+^CD3^−^RANK^+^IL-7Rα^hi^ lymphoid tissue inducer (LTi) cells [known also as type 3 innate lymphoid cells (7–9)]. These express retinoic acid-related orphan receptor-γt (ROR-γt) and lymphotoxin LTα_1_β_2_; a heterotrimeric complex that comprises membrane-bound LTβ and soluble LTα; its binding to lymphotoxin-β receptor (LTβR) on VCAM-1^+^ ICAM-1^+^ LTβR^+^ stromal tissue organizer (LTo) cells establishes a lymphoid chemokine feedback loop, involving CC-chemokine ligand 19 (CCL19), CCL21, and CXC-chemokine ligand 13 (CXCL13), which in turn drives early B/T cell clustering and segregation as well as the differentiation of high endothelial venules (HEVs) (10–14). Although the organogenesis of the spleen, LNs, and Peyer’s patches clearly requires LTi cells (11, 15, 16), there remains uncertainty about the identity of the equivalent cells that prompt TLO induction.

CD4^+^CD3^−^ LTi cells are present in adults, albeit at much lower frequency (17, 18), express lymphotoxin (LT) and tumor necrosis factor (TNF) (19) and provide support to T follicular helper (T_FH_) cells in GCs (20), as well as contributing to memory humoral immune responses (21). Unlike embryonic LTi cells, adult CD4^+^CD3^−^ cells express high levels of OX40L and CD30L (22). LTi cells may be similarly involved in TLO formation (23) but there is conflicting evidence to support this hypothesis. First, interleukin-7 (a key survival factor for LTi cells in developing SLOs) transgenic mice develop organized TLOs after immunization with antigen, in a process that is dependent upon LTα_1_β_2_ and the LTi-associated transcription factor retinoic acid-related orphan receptor-γt (ROR-γt) (24). Second, intra-dermal injection of newborn mesenteric LN-derived cells (containing stromal organizer cells and LTi cells but not mature lymphocytes) into adult mice can induce formation of lymphoid tissue in the skin, with the aggregates composed of donor-origin stromal cells and recipient-derived lymphocytes organized into distinctive areas (25). And third, overexpression of CXCL13 in non-lymphoid tissue, such as the pancreas results in TLOs containing B and T cell zones, HEVs, and stromal cells (26); pancreatic tissue in these transgenic animals contain a significant population of CD4^+^CD3^−^IL-7Rα^hi^ cells, suggesting that chemokine driven LTi-type cells expressing LTα_1_β_2_ (27) may play a role in the formation of ectopic lymphoid tissue as well as native SLOs (26–28). Nevertheless in transgenic models of TLO formation, ectopic lymphoid neogenesis has started before birth and it is possible that *de novo* TLOs established as a result of chronic inflammation are different to developmentally programmed TLOs in their requirement for LTi cells.

There is, however, also evidence that TLOs can form in the complete absence of LTi cells. For instance, mice deficient in the nuclear hormone ROR-γt and the transcriptional repressor Id2 still can still form intestinal TLOs in response to microbiota, despite lacking LTi cells (29). Similarly, Marinkovic et al. showed that formation of TLOs in thyroid tissue occurs by mature CD3^+^ CD4^+^ T cells, and not by LTi cells, and that these cells promote ectopic HEV development by LTβR signaling (30).

**Figure 1:**
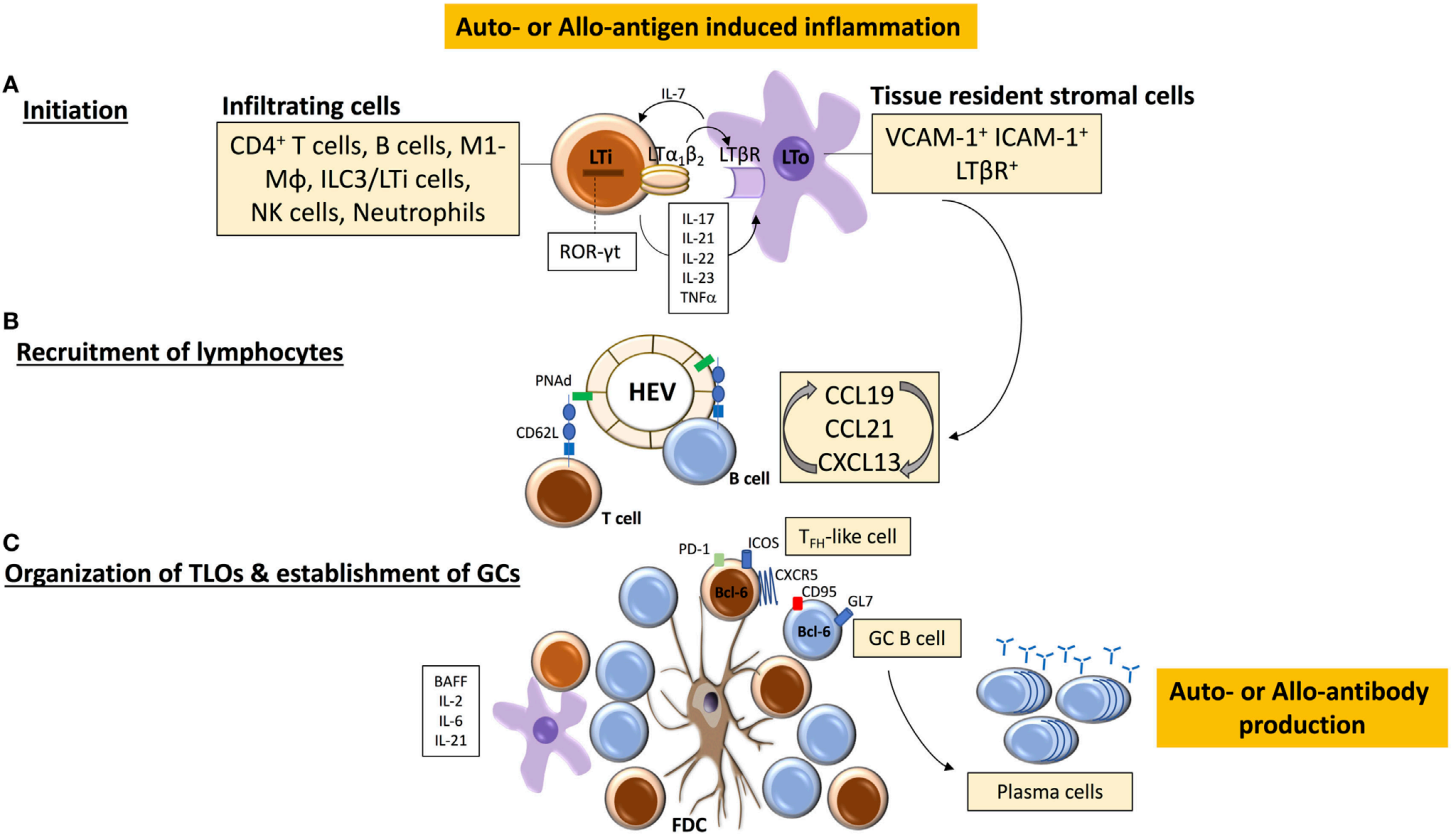
Tertiary lymphoid organ (TLO) initiation and formation. **(A)** TLO-initiating immune cells [among which are lymphoid tissue inducer (LTi)-like cells] accumulate at sites of inflammation and interact with stromal mesenchymal lymphoid tissue organizing (LTo) cells. The binding of LTα_1_β_2_ on LTi cells with LTβR on LTo cell leads to the release of chemokines CCL19, CCL21, and CXC-chemokine ligand 13 (CXCL13) that mediate further immune cell recruitment and spatial organization within the forming TLO. **(B)** Similarly, local release of homeostatic chemokines drives the formation of high endothelial venules (HEVs) and lymphangiogenesis, leading to homing of (auto-or alloreactive) naïve and memory B and T cells. A well-organized TLO is composed of compartmentalized T and B cell areas, follicular dendritic cells (FDC), dendritic cells, HEVs, and lymphatic vessels. **(C)** Under the influence of LTα_1_β_2_, stromal cells acquire the phenotypic and functional properties of FDCs, which facilitate persistent antigen presentation within TLOs, and CD4^+^ T cells acquire follicular helper (T_FH_)-like effector characteristics (CXCR5^hi^PD-1^hi^ICOS^hi^) to drive activation of B cells. Cytokines, such as B-cell-activating factor (BAFF), IL-21, and IL-6, contribute to the survival and maintenance of T_FH_ cells and germinal center (GC) B cells, which subsequently differentiate into antibody-secreting plasma cells.

One of the main questions, therefore, is what cell type(s), equivalent to LTi and LTo cells for SLO development, drive(s) TLO formation (Figure 1). Since TLOs arise postnatally in response to inflammatory triggers, immune cells may substitute for LTi cells and act as the primary initiators of tertiary lymphoid neogenesis. Analysis of explanted allografts due to chronic rejection has shown that the development of TLOs depends upon the recapitulation of the genetic programme fundamental to the development of SLOs (31). When the reprogramming is incomplete, only naïve B cell clusters form, whereas if the recapitulation is complete, functional ectopic GCs generating anti-HLA secreting plasma cells develop. This implies that the mechanistic pathways involved in SLO and TLO formation are very similar; as confirmation, we have also shown that LT signaling is essential to the formation of TLOs in chronically rejecting allografts (32). The suggestion that persistent antigen exposure is critical for maintaining TLO organization is supported by the finding of secondary B cell follicles with GCs and only rare primary B cell follicles in chronically inflamed tissues (in autoimmune disease), and by the finding that ectopic (autoimmune) GCs generate plasma cells that produce antibodies specific for antigens that are expressed in the target tissue (33, 34).

Lymphotoxin expressing cells other than LTi cells can drive TLO formation, such as M1-polarized pro-inflammatory macrophages (35), and T (36) and B cells (29) which upregulate LTα_1_β_2_ expression in response to ectopic expression of CCL21 and CXCL13, respectively (37). The central role of B cells in initiating allograft-TLO formation would seem to be supported by experimental and biopsy-based studies within the last decade showing that TLOs within kidney, heart, or lung grafts are predominantly composed of B cell clusters organized into follicles, segregated from T cell and plasma cell areas (32, 38–44). Further analysis has revealed that TLOs can closely resemble a classical secondary follicle, consisting of proliferating (Ki67^+^) B cells in close proximity to CXCL13 and supported by a network of follicular dendritic cells (FDCs), surrounded by naïve follicular mantle (IgD^+^) B cells (45–49). Such ectopic GCs have been consistently found within chronically rejecting allografts (32, 40, 45) and have been identified in autoimmune-associated TLOs within peripheral tissues (50–52). A LTαβ-dependent LTi-like role for B cells in the development of TLOs has also been described in dextran sulfate sodium-induced colitis (29).

What, however, governs the infiltration and survival of B cells in allografts and drives them to form TLOs? Recipient-derived B cells and B cell clusters (along with B cell transcripts) have been found in acute and chronically rejecting solid organ allografts where they can contribute to both humoral and cellular allograft rejection (53–56). Chemokines and chemokine receptors are critical in leukocyte recruitment, activation, and differentiation, with CXCL10 ligand and its receptor CXCR3 (57) as well as CXCL13 and CXCR5 (58) and chemokine receptor CCR1 (59) governing the recruitment of B cells. Gene expression profiling of renal allograft biopsies has shown that expression of these chemokines plus their corresponding receptors is strongly correlated with rejection than with stable allograft function (60). A recent meta-analysis has shown that the presence of CD20^+^ B cell infiltrates within allografts was correlated with more aggressive and steroid-resistant graft rejection and with an increased risk of graft loss (61). Zarkhin and Sarwal performed immunostaining on transplant nephrectomy microarray samples for B cell phenotypes and found two lineages of B cells: interstitial CD20^+^ B cell clusters, most of which had an activated phenotype as they were positively stained with CD79a, and scattered CD38^+^ plasmablasts and plasma cells (53, 62). The B cell clusters also stained for MHC class II (HLA-DR) and were surrounded by CD4^+^ T cells, suggesting a role for local antigen presentation by B cells. The antigen-presenting function of infiltrating B cells (63) might contribute to an augmented alloreactive response and thus drive aggressive T cell-mediated cellular rejection by activating alloreactive T cells into effector and memory cells (40, 64). This hypothesis is supported by studies showing a strong correlation between scattered CD3^+^ T cells and CD20^+^ B cell infiltrates in renal allografts during acute rejection (65). These B cell infiltrates within the transplant can then initiate formation of intra-graft TLOs. It should be noted, however, that other studies that have failed to show a correlation between CD20^+^ B cell infiltration or immunoglobulin transcript expression in acute rejection and poor late graft function (56, 65) implying that B cells and plasma cells can be recruited and retained in inflammatory compartments in allografts as a nonspecific feature of chronic inflammation. The lifespan of antibody-secreting plasma cells depends upon residence in a survival niche, such as the bone marrow and to a lesser extent, the red pulp of the spleen (66, 67). In conventional immune responses, the migration of post-GC plasma cells from SLOs to the bone marrow depends on the interaction between CXCR3 and CXCR4 on plasma cells and CXCL9, CXCL10, and CXCL12 produced by bone marrow stroma (68, 69). Inflammation within an allograft can similarly create a chemokine and cytokine rich niche (60) to support long-term plasma cell survival, so it is, therefore, possible that intra-graft plasma cells originate within conventional lymphoid tissue rather than within TLOs and migrate to the inflammatory milieu within the allograft, as has been described for plasma cells within inflamed kidneys in a mouse model of Systemic Lupus Erythematosus (70). However, given the intimately close proximity of plasma cells to ectopic GCs (31, 32, 43, 71, 72), this possibility would seem to be less likely.

Evidence to support the potential role of B cells in TLO formation has come from studies examining their role in the maintenance of SLO architecture in adults. LTβ expression can be upregulated in B cells following antigen engagement and by chemokine CXCL13 signaling (73), and it has been shown that the absence of LT-expressing B cells results in the inability of isolated lymphoid follicles (B cell follicle-containing lymphoid structures along the length of the mesenteric wall of the small intestine) to develop fully, suggesting that B cells participate in the development of mucosal lymphoid tissue (74). B cells have been shown to deliver the signals necessary for both the maintenance of lymphoid follicles within SLOs in adult animals as well as the maturation of FDCs (75). It was initially unclear if naïve B cells can express LTα_1_β_2_ (76, 77). Although naïve B cells express LTβ constitutively (78), expression of the LTα subunit requires stimuli such as CD40L (79), IL-4 (79) or endotoxin (80). Similarly, LTα expression on T cells is upregulated after activation of the T cell receptor by anti-CD3 and by CCL21 (37, 81). The role of B cells in this process has been further dissected by preventing B cells from expressing LTα_1_β_2_, by selectively deleting the LTβ gene in B cells (B-LTβ knockout mice) (82). Splenic B cell follicles and FDC networks are disrupted in these animals, and even though GCs can develop in response to antigenic stimuli, albeit somewhat reduced in size and number, IgG responses are impaired. These findings imply that B cells, *via* membrane-bound LTα_1_β_2_, transmit signals required for the development and function of stromal cells that produce chemokines essential for normal SLO organization, and so could also extend to B cell involvement in initiating TLO formation.

A further question to consider is the profile of adult lymphocytes that initiate and maintain TLOs—are they naïve or activated cells? One of the hallmark characteristics of SLOs is their exquisite ability to recruit circulating naive lymphocytes and elicit priming and subsequent clonal expansion of antigen-specific T and B cells (1, 83). In general, naive lymphocytes circulate among SLOs; in LNs this occurs *via* binding of L-selectin/CD62L with a family of mucin-like sulfated glycoproteins, also known as peripheral node addressins (PNAd), on HEVs (84), resulting in lymphocyte rolling on the endothelium, which represents the first step in homing to LNs. Antigen-experienced (primed) lymphocytes, however, as a result of interaction with antigen-presenting cells, modify their expression of adhesion molecules and chemokine receptors (e.g., CD62L and CCR7) and have different migratory patterns (85). The expression of specific combinations of these receptors allows primed lymphocytes, including memory T cells, to interact with blood vessel endothelium and to migrate into peripheral tissues for responses to inflammatory stimuli (86). Whether TLOs maintain an immune response that originates from circulating activated B and/or T cells and/or from naive cells remains to be fully defined. As HEVs are a fundamental constituent of conventional LNs that permit naive lymphocyte egress from the circulation for adaptive immune responses (87), HEVs in allograft-TLOs (32, 39, 88) could have important pathological consequences, as they could facilitate entry of naïve T and B cells (previously excluded by the absence of cognate ligands for CCR7 and L-selectin) and also central memory T cells, by way of their expression of CD62L (89), and allow an alternative site for lymphocyte priming, activation, and effector function (64). However, it is more plausible that the initial ingress of lymphocytes that establish TLOs along with HEVs comprise an effector population; in support the majority of the infiltrating B cells in renal allografts display activated (CD79^+^) and memory (CD27^+^) phenotypes (62). From our experimental work, we have shown that fully mature allograft-TLOs fail to form in B cell deficient animals; ectopic lymphoid aggregates do form (presumably a consequence of the cellular alloimmune response) but were not well circumscribed and importantly lacked HEVs, implying that B cells are critical for TLO formation but as an activated, rather than naive, population (32). Therefore, it is likely that naïve recipient B cells, initially primed by alloantigen in SLOs, migrate to allografts after altering their expression of adhesion molecules and interact with resident (recipient) stromal cells to establish of a chemokine-directed positive feedback loop that orchestrates further lymphocyte recruitment and organization and formation of HEVs (73). B cells, upon sustained antigenic challenge in chronically inflamed tissues, can upregulate LT expression through IL-4Rα signaling and promote the proliferation and activation of supporting fibroblastic reticular cells (FRCs) *via* LTβR signaling (90). FRCs can in turn secrete T cell chemokines, generate support structures for migrating T cells and dendritic cells (91) and form a conduit to distribute small soluble antigens throughout lymphoid organ parenchyma (92, 93). Moreover, FRCs enhance the survival of naive T cells by producing IL-7 (94), present antigen to T cells (95), and support the differentiation of regulatory dendritic cells (96). As a result, lymphocytic infiltrates in chronically inflamed tissues can eventually acquire the structural characteristics to develop into a lymphoid organ to support further lymphocyte recruitment and retention (see also Figure 1). In addition to the role of lymphoid chemokines, cytokines produced within the inflammatory milieu of an allograft or peripheral organ affected by autoimmunity support the development of TLOs and compartmentalization into T and B cell areas, and can also contribute to the transcriptional regulation of CXCL13, CCL19, and CCL21. Of note, IL-17, IL-21, IL-22, IL-23, and TNF have all been shown to be important (36, 97–103), with the level of certain pro-inflammatory cytokines correlating with disease outcomes (104).

### The Other Cast Members for TLO Initiation—T Cells and Innate Immune Cells

Besides B cells, a variety of T cells and innate immune cells are also involved in ectopic lymphoid neogenesis independent of lymphoid-tissue inducer cells. In particular, Il-17 producing CD4^+^ Th17 cells have been shown to be essential for the formation of TLOs in the central nervous system of mice during chronic experimental autoimmune encephalomyelitis (EAE), where stromal LTβR signaling promoted extracellular matrix deposition, T cell effector cytokine responses, and chemokine production that supported meningeal leukocyte accumulation (105), and for responses against collagen (V) protein to induce the development of bronchiolitis obliterans after lung transplantation (106). Furthermore, there is a correlation between activation-induced cytidine deaminase (AID)-expressing ectopic GCs within chronically rejecting kidney allografts and the cytokine IL-21, suggesting that Th17 cells have a role to play in lymphoid neogenesis (107). IL-21 secretion from Th17 cells (108) can stimulate B cells through the generation of T_FH_ cells (109, 110), the importance of which will be discussed in a later section.

A key player in innate immune responses are macrophages and not only can they act as antigen-presenting cells within TLOs (111), and produce CXCL12 to drive migration of CXCR4^+^ centroblasts to the dark zone in ectopic GCs (99), they can also initiate TLO formation; in particular, M1-polarized pro-inflammatory macrophages can substitute for LTi cells and trigger chemokine expression by vascular smooth muscle cells (similar to that produced by LTo cells) independently of LTβR signaling (35). Monocytes and macrophages also secrete CXCL13 in TLOs associated with rheumatoid arthritis and ulcerative colitis (112), providing a rich source of lymphoid chemokines in chronic inflammation. CXCL13 can be expressed within and near smaller collections of B cells in diseased tissue where no FDCs or HEVs are detected, suggesting that CXCL13 production by monocytes could be an early event in lymphoid neogenesis. Similarly, accumulations of macrophages within the lumens of capillaries and small vessels of the myocardial interstitium are a prominent feature of cardiac allograft antibody-mediated rejection (AMR) (113) and tubulointerstitial cellular rejection (Banff category 4, type I) in renal allografts (114), with presence of macrophages in early biopsies predictive of interstitial fibrosis/tubular atrophy and subsequent graft failure (115–117). Besides acting as a source of CXCL13 to promote development of CXCR5^+^ B cell aggregates in chronically rejecting cardiac allografts (46), infiltrating macrophages can also transdifferentiate into lymphatic endothelial cells or secrete vascular endothelial growth factor to drive the growth of lymphatic vessels (LVs) through the sprouting of preexistent lymphatics (118, 119). The significance of lymphangiogenesis in transplantation was shown in a study by Kerjaschki and colleagues who identified vast amplification of LVs near or within TLOs in renal allografts with acute rejection (41).

### The Counterpart to the Initiators—Stromal Organizer Cells

The counterparts to the initiators cells in the development of SLOs are stromal organizer cells (10). LTi cells, through expression of surface LTα_1_β_2_, activate mesenchymal stromal cells *via* LTβR signaling to express adhesion molecules, such as ICAM1, VCAM1, MAdCAM1, and PNAd, and a set of homeostatic chemokines (CCL19, CCL21, CXCL12, and CXCL13), which then allows them to be retained in the developing organs and will regulate further lymphocyte homing and compartmentalization (13). After birth, LTo cells undergo further differentiation into various non-hematopoietic stromal subtypes present in the adult SLO, such as FRCs of the T cell zone, FDCs within B cell follicles and GCs, and marginal reticular cells adjacent to the subcapsular sinus (120). LN stromal endothelial cells can differentiate into either blood endothelial cells (HEVs) or lymphatic endothelial cells (121). Subsequently, these structures are colonized by lymphocytes resulting in a highly organized lymphoid organ.

Stromal organizer cells are mesenchymal in origin, with many cell types capable of playing this role (122); these include fibroblasts, pericytes (a type of smooth muscle cell commonly found surrounding capillaries and HEVs in LNs), blood and lymphatic endothelial cells, and epithelial cells. Stromal cells also play a critical role in TLO formation in that they provide an environment that is conducive to lymphoid neogenesis (25, 123–126); their characteristics and function have been the subject of two recent comprehensive reviews (127, 128). TLOs commonly arise close to vascular or epithelial ductal structures adjacent to smooth muscle cells or myofibroblast-like cells that share features with conventional LTo cells, e.g., synovial fibroblasts from patients with rheumatoid arthritis display LTo-like properties, including expression of LTβR and the production of homeostatic chemokines (e.g., CXCL13) (129) and B-cell-activating factor (BAFF) to support synovial B cell responses (130), and LTβR signaling has been shown to induce aortic smooth muscle cells to form TLOs in atherosclerosis (71). Nevertheless, LT signaling is not absolutely required for activation of stromal cells and leukocytes other than lymphocytes can activate resident tissue fibroblasts (126). Moreover, it is also possible that circulating fibrocytes may be recruited by homeostatic chemokines to sites of TLO development (131). It has subsequently been proposed that stromal cell activation in TLO formation is a two-step process: the inflammatory cytokine milieu initially primes local resident stromal cells independent of LT signaling and maturation to LTo-like cells occurs as a result of signaling by LT-expressing immune cells, resulting in the generation of homeostatic chemokines that promote lymphocyte compartmentalization (127, 128). The activated stromal cells can also influence the type of immune responses; for instance, activated fibroblasts can secrete IL-6 which is required for the induction and maintenance of T_FH_ cells (132) and can induce T cell tolerance by presenting high levels of peptide-MHC class II complexes (133), thus suggesting an immunoregulatory role for stromal cells in the context of TLO development.

### HEVs—A Vascular Component of the Stromal Network

High endothelial venules are an integral part of the stromal network of LNs and as mentioned above they facilitate the trafficking of naïve recirculating B and T lymphocytes from the circulation. HEVs are prominent features of TLOs and their presence can be considered the defining characteristic that distinguishes lymphocyte aggregates from other forms of inflammatory infiltrate (39). The characteristics of HEVs in TLOs have been extensively reviewed elsewhere (88, 134), and although HEVs in TLOs express the same chemokines [e.g., CCL19, CCL21 (135)], adhesion molecules [e.g., ICAM-1 (72)], and ligands [e.g., PNAd (32, 136) and MAdCAM-1 (32, 72)] as those in conventional LNs, it is important to point out that the actual migration of intra-vascular naïve lymphocytes from the systemic circulation via HEVs in allograft-TLOs into the parenchyma of an organ has not yet been visualized. The development of mice with red fluorescent LVs and green fluorescent HEVs (137) and their *in vivo* imaging in SLOs (138) will enable similar analysis in TLOs and resolve whether HEVs function as sites of entry for naïve alloreactive lymphocytes into ectopic lymphoid tissue to undergo activation and differentiation into effector and memory cells.

The precise mechanisms and signaling molecules besides LTα_1_β_2_ that drive stromal cell activation and differentiation in TLOs are not yet fully defined and the identity of LTo cells remains somewhat elusive as they lack specific markers and so have not yet been isolated from TLOs. Several questions also remain unanswered. First, do tissue stromal cells convert to a “lymphoid-like” phenotype as a result of inflammation (126), or do they arise in TLOs from progenitors such as mesenchymal stem cells? Second, is the chemokine expression by stromal cells a result of ingress of LT-expressing (alloreactive) lymphocytes or does the lymphoid stroma undergo expansion prior to the infiltration of lymphocytes? Third in the context of transplantation, do stromal cells arise from resident cells in the allograft or are they derived from the recipient? And fourth, what signaling pathways can be targeted to manipulate TLO stromal cells? Transplantation provides a unique opportunity to answer some of these questions as based on the above it would seem likely that recipient-derived lymphocytes interact with donor derived stromal cells to establish TLOs. This could be analyzed by selectively ablating donor-derived stromal cells without impacting lymphoid stroma in (recipient) SLOs, for example by using donors lacking expression of LTβR (139) or by conditionally depleting fibroblasts in allografts either based on their expression of the diphtheria toxin receptor (140) or by using inducible transgenic mice [*Ccl19*-Cre × iDTR] (141).

## The Functional Significance of the B Cell Component within SLOs

As discussed above, the prominence of follicular structures within the TLO may reflect an integral role for the B cell in its formation, most likely as a source of membrane-bound LT (142, 143). Nevertheless, the presence of B cell follicles raises important questions relating to the function they provide; most critically, whether the humoral response within the TLO is simply an extension of those occurring synchronously in SLOs or whether it provides unique and distinct capabilities. If the former, a simple consideration of the respective volume of lymphoid tissue within the TLO and SLO would suggest it unlikely that the TLO response will materially impact upon either the nature or strength of the global humoral response. “Distinctiveness” of the humoral TLO response is likely determined by the nature of the GC component, because of the more sophisticated output—the production of affinity-matured memory B cells and LLPCs—that the GC generates (144). In this respect, while it is clear that immunohistochemical features of the GC response, such as expression of AID and presence of FDCs and GC-phenotype B cells, have been described within TLO (32, 71, 145–157), important differences from conventional (SLO-resident) GC appearances have also been noted (discussed below).

Over the last decade, a number of seminal publications have substantially improved our understanding of the conventional GC response [reviewed in Ref. (158–163)]. By detailing some of the molecular pathways responsible for coordinating the GC response, these publications have reinforced that the anatomical constraints, namely the physical segregation of the GC into light and dark zones, are integral for GC function. The greater insights provided by these studies, thus, enable a timely re-appraisal of the likely function of the GC response within TLOs. To do so, it is first necessary to consider how our understanding of the GC response has evolved.

### The Initiation and Maintenance of Conventional GC Responses within SLOs

Following encounter with target epitope, antigen-specific T and B cells migrate to the T–B cell border (164) or interfollicular zone (165) as a consequence of alterations in sensitivity to the CCR7 ligands, CCL19, and CCL21 (166). Following robust proliferation, responding B cells either seed the extrafollicular response in LN medullary cords and the red pulp in the spleen (167), or a relatively small proportion migrates back to the follicle to seed the GC response (168). Retention within the follicle is maintained by downregulating expression of the orphan G protein-coupled receptor Ebi2 (169, 170) and induction of the sphingosine 1-phosphate receptor, S1P2 (171, 172). The determinants for clonal selection to the follicle remain unclear, because although it has been reported that high-affinity clones may have a selection advantage (173), this has been similarly proposed for the extrafollicular focus (174). Analysis, moreover, of hyper-mutated broadly neutralizing antibodies to HIV has highlighted that the germline configuration may have only minimal reactivity (175). Two recent publications have instead suggested that seeding of the GC is largely stochastic (176, 177).

The small number of clones that seed the nascent GC become a foci of proliferating blasts leading to the formation of the archetypal light and dark zones, first identified by light microscopy over 80 years ago (178). Within the dark zone, CXCR4^hi^CD83^lo^CD86^lo^ “centroblasts” are retained by CXCL12-expressing reticular cells (179–181) and undergo between, typically, one and six rapid divisions (182, 183). Expression of AID and upregulation of Polη DNA polymerase introduces point mutations into the genes encoding the B cell receptor (BCR) (180, 184): the dark zone is, therefore, the region where immunoglobulin somatic hypermutation (SHM) occurs. Upon egress to the light zone, facilitated by a chemokine gradient toward CXCL13-expressing FDCs, “centrocytes” acquire an activated CXCR4^lo^CD83^hi^CD86^hi^ phenotype and reset their antigen-processing machinery (185), prior to encounter with antigen on the surface of the FDC.

Within the light zone, several different outcomes are possible for the centrocyte: death from apoptosis; differentiation to a memory B cell or to a LLPC; and re-entry into the dark zone for a further round of mutation and selection. Although many aspects of this process remain poorly understood, it is now clear that the specialized T_FH_ subset (186, 187) is critical for selection of high-affinity variants within the GC light zone and is the driving force of affinity maturation (158–160, 162). T_FH_ cell differentiation is initiated by EBi2-guided recognition of target epitope on dendritic cells in the outer T cell zone, with “quenching” of T cell IL-2 by CD25 on the DC possibly prompting expression of Bcl6 (188, 189), the master transcription factor for T_FH_ differentiation (190–192). Subsequent cognate interaction of the pre-T_FH_ cell with the antigen-specific B cell at the T–B cell border, along with co-stimulatory signals delivered through CD28, OX40, and ICOS (193–195), is generally required to complete T_FH_ cell differentiation and is critically dependent upon prolonged association between SLAM and SAP family members (196–200): in the absence of SAP, T_FH_ do not form. Interestingly, adult CD4^+^CD3^−^ cells residing at the border of the T cell zone and B cell follicles have been shown to express OX40L, which helps direct OX40-expressing pre-T_FH_ cells into the follicles and induces upregulation of CXCR5 and ICOS expression (20, 194, 201). CXCR5^hi^PD-1^hi^ICOS^hi^ CD4 T_FH_ cells are then guided to the B cell follicle under the influence of CXCL13 and EBi2 gradients (202). Migration occurs 1 or 2 days later than the B cell (165, 203, 204) and of note, this migration is also dependent upon ICOSL signaling from bystander B cells within the follicle; in the absence of this signaling, T_FH_ cell migration to the follicle is substantially impaired (205).

A number of recent studies have confirmed the pivotal role that the T_FH_ cell plays in affinity maturation of the antibody response (179, 180, 182, 183, 203, 204). B cells internalize antigen *via* their BCR for presentation to the helper T cell in an affinity-dependent manner (206–208), and hence, somatically mutated B cells that have acquired greater amounts of target antigen from the light zone FDC can outcompete other clones for the limiting help available from the GC T_FH_ subset (180). These clones can then return to the dark zone for further rounds of mutation and selection. The nature of the signal that the T_FH_ provides to the B cell remains unclear, and likely involves IL-21 (209) and BAFF (210), resulting in both an avoidance of apoptosis within the light zone, as well as a subsequent selection advantage within the dark zone (179, 182, 183). Thus, availability of T cell help, rather than access to antigen on the FDC, determines selection of high-affinity variants. From this, one would assume that the T_FH_ cell subset would encompass those responding T cell clones with highest affinity for the peptide complexes presented by the B cell (211, 212). However, Shulman et al. have recently suggested that selection into the T_FH_ cell population is more permissive, without apparent clonal restriction (203).

Although our understanding of clonal selection within the GC has improved substantially in recent years, with the functional relevance of the GC being primarily a producer of LLPCs and memory B cells, it is perhaps surprising that the triggers governing the generation of these effector populations remain unclear. The progeny of a single B cell can differentiate to seed all the GC and post-GC compartments (213), but LLPCs, first evident as BLIMP-1 expressing “pre-plasma cells” within the GC (214, 215), appear to be selected actively from the highest-affinity variants (216–218). Plasma cell differentiation is informed by the signaling tail of the particular immunoglobulin isotype (219), and although it can occur in the absence of T cell help (220), CD40 signaling from cognate interaction with the helper T cell also appears important (221, 222). Krautler et al. have recently proposed that plasma cell differentiation is initiated by high-affinity contact with target antigen, but is thereafter dependent upon receipt of T cell help (223). By contrast, memory B cells are deposited early, and then continuously, from the GC reaction (218, 224–226). Consequently, compared to the contemporaneous GC B cell, the memory B cell pool exhibits less extensive SHM and its binding affinity for target antigen is much weaker than is observed within the plasma cell output. Memory B cell precursors may arise from low-affinity variants within the light zone, and Shinnakasu et al. have recently reported an inverse correlation with the level of T cell help delivered and *Bach2* expression within the B cell, and have suggested that relatively high *Bach2* expression within lower-affinity centrocytes favors entry to the memory B cell pool (227).

### GC Responses within TLOs

It is, therefore, clear that the GC reaction is remarkably complex, and that its effector functions are dependent upon tight anatomical and temporal control, as evident from the association between dysregulated GC responses and development of humoral autoimmune disease (228–230). This raises the critical questions of whether such sophistication can be recapitulated within an ectopic GC in a peripheral organ, and whether it would match concurrent selection within conventional SLO. This has proven difficult to address experimentally, and although a number of publications have reported anatomical features consistent with GC activity, such as the presence of FDC and AID-expressing B cells with GL7^hi^ GC immunophenotype (see Table 1), few studies have attempted to validate GC activity by analyzing effector output. The most robust indicator of GC effector function will be the demonstration of clonal selection with progressive accumulation of high-affinity mutants as a consequence of SHM. It should be stressed that simple clonal restriction within a TLO, whereby TLOs harbor a limited number of B cell clones and these clones differ between individual TLOs, may not itself indicate GC activity; rather it may simply reflect focused seeding of the TLO by locally activated B cells, as reported by Scheel et al. (153). Evidence for SHM within the TLO is, thus, provided by only a handful of studies (146, 154, 157), with possibly the most convincing a relatively early study by Stott et al. (154), who performed Ig V-region sequencing of B cells recovered by laser microdissection of salivary gland TLOs in human patients with Sjogren’s syndrome. Although specific target antigen was not identified, sequencing analysis of the ratio of replacement to silent mutations in the complementarity determining region suggested antigen-mediated selection of high-affinity variants.

**Table 1:** Studies characterizing germinal center (GC)-like structures within tertiary lymphoid organs (TLOs).

	System	Structure and cells				Diverification		Notes
	Species/organ	High endothelial venules (HEV)	Follicular dendritic cells (FDC)	B cells/GC markers	T cells/T_FH_	Evidence of somatic hypermutation (SHM)	Increased mRNA/protein expression	
Motallebzadeh et al. (32)	Murine/heart Tx	Y	Y	B220/PNA	NR	NR	NR/activation-induced cytidine deaminase (AID)	Blocking lymphotoxin (LT) signaling by LTβR-Ig impairs TLO development and effector antibody response
Thaunat et al. (31)	Human/kidney Tx	Y	Y	CD20/BcL-6	CD3	N	AID, CXC-chemokine ligand 13 (CXCL13), CXCR4, CC-chemokine ligand 19 (CCL19), CCL21, CCR7, LTα, LTβ, and CXCR5	Extensive characterization of gene expression involved in lymphoid oranogenesis
Grabner et al. (71)	Murine/ApoE Aorta	Y	Y	B220/Ki-67	CD3	NR	CXCL13, CCL21, Ltb	CD138^+^-Plasma cells, Tregs No antibody characterization
Clement et al. (145)	Murine/ApoE aorta Human/AAA	NR Y	NR Y	B220/CD95^hi^ CD20/PNA	CD4/CXCR5^+^PD-1^+^ CD4/CXCR5, ICOS, PD-1	NR NR	NR NR	CD8^+^ regulatory T cells regulate secondary lymphoid organ (SLO) and TLO responses
Vu Van et al. (146)	Murine/iBALT	NR	Y (very low)	CD19/GL7, PNA, Bcl6, Cd38^lo^	Th-like but no CXCR5^+^BCL-6^+^T_FH_	Yes	NR	FDC not associated with GC B cells; SHM (NP as target), but no comparison to SLO High CD138 plasma cells
Germain et al. (147)	Human/lung tumor		Y	CD20/Ki-67, Bcl-6	CD3	NR	AID	Described CD23^+^ mantle zone, CD138 plasma cells at the periphery of TLO B cell density in TLOs as prognostic biomaker
Martinet et al. (231)	Human/solid tumors	Y	NR	CD20/NR	CD3	NR	CCL19, CCL21, CXCL13, and CCR7	Large-scale FACS analysis on immune populations retrieved from TLOs revealed that tumor HEVs are associated with increased numbers of effector (cytotoxic, and memory) and naïve T cells
Cipponi et al. (232)	Human/melanoma	Y	Y	CD20, CD138, AID, Ki-67	NR	Yes	AID	While cutaneous metastic lesions contained TLOs, primary melanomas lacked B cell clusters but contained HEVs. TLO-derived Ig gene repertoire demonstrated clonal amplification, SHM, and isotype switching
Coppola et al. (233)	Human/colorectal cancers (CRC)	NR	Y	CD20/Ki-67	CD3	NR	CCL2, CCL3, CCL4, CCL5, CCL8, CCL18, CCL19, CCL21, CXCL9, CXCL10, CXCL11, and CXCL13	Extensive metagene analysis using gene chip technology, and 12-chemokine gene screening was performed on 326 CRCs suggested that TLO structures are associated with better prognosis
de Chaisemartin et al. (234)	Human/lung tumor	Y	Y	CD138	CD4, CD62L	NR	CCL19, CCL21, CXCL13, CCL17, CCL22, IL-16, ICAM-2, ICAM-3, ICAM-1, and MadCAM-1	Characterization of adhesion molecules and chemoattractants of lymphoid cells into lung cancer tissue
Slight et al. (148)	Murine/lung TB		Y	B220/PCNA, PNA	CD4/T_FH_	NR	CXCL13	TLO contained Th1-like cells; CD4^+^ CXCR5^+^ T cells is essential for TLO localization
Bombardieri et al. (149)	Murine/SS induction *via* ADV5 delivery	Y	Y	B220/GL7	CD3	NR	AID, CXCL13/CXCR5, CCL19/CCR7, and LT-β	No antibody characterization
Gu-Trantien et al. (150)	Human/breast cancer	NR	Y	CD20/Ki67	CD4/T_FH_ phenotype	NR	CD200, CXCL13, ICOS, PD1	TLO contained Th1, Th2, Th17, Tregs, and memory T cells
Nacionales et al. (151)	Murine/TMPD lipo-granuloma	NR	N	B220/Ki-67	CD3	Y	AID expression splenocytes > TLO > peritoneal exudate cells	Antigen-driven SHM CSR (excision circles)
Cheng et al. (152)	Human/kidney Tx	NR	NR	CD20	NR	Y	*Rag-1, Rag-2*	Antigen-driven SHM; clonal restriction in peripheral, and TLO-derived Ig genes
Scheel et al. (153)	Human/RA B cells synovial fluid	N	N	N	NR	Y	NR	Synovial fluid lacked GC formation, but contained B/T cells aggregates. Plasma cells aggregates are the consequence of migration of plasmablasts from peripheral lymphoid organs
Stott et al. (154)	Human/SS salivary glands	NR	Y	CD20	CD3	Y	NR	Important reported LZ/DZ, large numbers of plasma cells in surrounding tissue
Grewal et al. (155)	Murine/salivary gland inoculation with CMV	NR	Y	B220/GCT, GL7, PNA, Ki-67	CD4	Y	AID, CXCL13 (lymphoid neogenesis), syndecan-1, Blimp-1, PAX5	Possible LZ/DZ development but not formally addressed
Corsiero et al. (156)	Human/RA	NR	NR	CD20, CD138	CD3	Y	CXCRL13, CXCR5, LT-β	CD19^+^ FACS sorting of VH/VL sequencing reviled affinity maturation and clonal diversity
Weinstein et al. (157)	TMPD lipo-granuloma	NR	Y	NR	CD3, CD4	Y	NR	Affinity maturation possibly less within TLO, antigen-specific T cells, proliferation, and cytokine production

*Tx, transplantation; iBALT, inducible bronchus-associated lymphoid tissue; ApoE°, atherosclerosis-prone apolipoprotein E-knockout; AAA, atherosclerotic aneurysmal arteries; SS, Sjogrens syndrome; TMPD, tetramethyl-pentadecane; RA, rheumatoid arthritis; TB, tuberculosis*.

As discussed above, the T_FH_ cell subset is now known to play a pivotal role in selection of high-affinity variant GC B cells. The T_FH_ cell subset is likely to be similarly important for GC TLO function, and as with B cells in allograft-TLOs, associated T_FH_ cells are probably derived from peripheral effector populations (36), but they have not been identified routinely within ectopic lymphoid tissue (145, 148, 150, 157). Indeed, in a murine airway inflammation model, Vu Van et al. have recently highlighted the presence of an unusual T cell subset within foci of bronchus-associated lymphoid tissue (146). This subset did not express classical T_FH_ cell phenotype, but appeared in close cognate contact with the B cell fraction, and therefore may resemble a non-classical T_FH_ cell subset, such as the NKT cell subset described recently in relation to anti-lipid antibody responses (235, 236). Of note, the help provided by this “NKT_FH_” subset resulted in minimal deposition of LLPCs and suboptimal affinity maturation of the humoral response (235, 236). Thus, non-classical T_FH_ present with ectopic GCs may be inherently incapable of driving affinity maturation.

Nevertheless, the survival of alloreactive B cells within allograft-TLOs could be supported in particular by IL-21 derived from T_FH_ cells, akin to their function within canonical lymphoid tissue. In support of the role of T_FH_ cells in allograft-TLOs, Liarski et al. (237) conducted a cell distance mapping study to identify tissue resident PD1^+^ ICOS^+^ T_FH_ cell: CD20^+^ B cell pairs and showed that 80% of T cells with a T_FH_ phenotype were engaged in tight cognate interactions with B cells in renal allograft biopsies that displayed features of mixed T cell-mediated rejection and AMR; by contrast, only 15% of the T cells were similarly engaged in biopsies with pure T cell-mediated rejection. Of note, most B cells in the mixed rejection biopsies were spatially removed from the T_FH_ populations, suggesting that only a discrete population of alloreactive B cells is maintained by T_FH_ cells. In addition, the ICOS^+^-stained cells isolated by laser capture microdissection from mixed rejection samples showed high expression of BATF, a transcription factor necessary for GC formation and maturation of antibody-secreting B cells (238), but Bcl-6 and IL-21, which are critical for T_FH_ differentiation and function (190, 239, 240), were only highly expressed in the samples which had frequent T_FH_:B cell conjugates. It is, therefore, possible that the B cells that are juxtaposed to T_FH_ cells form TLOs and develop ectopic GCs, inducing Ig SHM and class switching to propagate local humoral alloimmune responses (146, 241, 242). The importance of T_FH_ cells is further evidenced by TLOs in autoimmune disease, where organized GCs containing peanut agglutinin binding GL-7^+^ B cells and ICOS^+^/CXCR5^+^ T_FH_ cells, along with CD138^+^ plasma cells, have been detected using laser capture microdissection and immunohistochemistry, with their presence correlating with tissue-specific autoantibody formation and progression of disease (33, 34). Blockade of T_FH_ cell infiltration by interrupting ICOS signaling results in reduced TLO formation associated with atherosclerosis as well as less severe disease progression (145). However, help provided by a classical T_FH_ cell subset within a TLO GC may not promote efficient affinity maturation. As demonstrated by the impaired affinity maturation that ensues when T cell help is artificially augmented (180), T_FH_ numbers must be closely controlled in order to maintain a competitive selection advantage for high-affinity variants. Whether this control is achieved within a TLO is not known, and it is perhaps more likely that help will be provided in a disorganized or dysregulated fashion. In support, in a model of tetramethylpecadentane-induced “lipogranulomas,” the presence of TLO was associated with, if anything, a reduction in affinity maturation (157). Similarly, loss of control of the T_FH_ cell subset within aortic TLOs exacerbates atherosclerosis (145).

Apart from T cell help, canonical GC responses are reliant upon FDCs, which can capture antigen–antibody complexes for presentation to B cells and express chemokines such as CXCL13, which draws B cells and T_FH_ cells to follicles *via* CXCR5 (243–246). FDCs also provide B cell survival and proliferation factors, such as BAFF (247). The BlyS family of TNF ligands (BAFF and APRIL) and their receptors [BR3 (also termed BAFF-R), TACI, and BCMA] govern survival and differentiation within B cell subsets, which is of particular relevance to humoral alloimmune responses (248). The receptors for BAFF are differentially expressed on B cells at various stages of maturation and activation, and will, thus, likely play a critical role in survival of B cells within TLOs. Thaunat’s group has shown that plasma cells which persisted within kidney allografts after administration of rituximab were intimately associated with BAFF secreting cells (249). As BAFF is mainly produced by macrophages, monocytes, and dendritic cells (250), the inflammatory microenvironment could, therefore, provide BAFF-dependent paracrine survival signals to intra-graft B cells in TLOs, as seen in B cell-rich lymphoid follicle-like structures in the meninges of EAE-affected mice where BAFF expression in inflamed tissues is upregulated in chronic relapsing forms of disease (52). Local BAFF could, therefore, provide additional survival signals for B cells within allograft-TLOs and promote tissue-resident humoral alloimmune responses as evidenced by an association between intra-graft BAFF and AMR in kidney transplantation (251), but also could protect autoreactive B cells generated during the (ectopic) GC response from apoptosis (see below) and sustain their differentiation into autoantibody-secreting plasma cells (252).

If not to promote affinity maturation, what role do GCs within TLOs perform? If one assumes that high-affinity mutants are not selected as effectively within a TLO as within a SLO, this implies that irrespective of similarities in clonal constituents at the onset of a response, the output from the SLO and TLO GC responses will increasingly diverge. The T_FH_ cell subset is, moreover, critical for ensuring negative selection within the GC—the destruction of potentially autoreactive variants that have arisen from SHM (253, 254). For example, in sanroque mutant mice, increased ICOS expression specifically on T cells results in an aberrant expansion of T_FH_ cells and spontaneous GC autoimmunity (255, 256). Conventional FoxP3 regulatory CD4 T cells (Tregs), as well as CD8 regulatory T cells (257, 258), are also necessary for prevention of humoral autoimmunity, likely through their inhibitory impact on the T_FH_ cell population. Thus, disorders in T_FH_ cell function (either intrinsic or through loss of external control) may allow autoreactive B cell variants within the TLO to escape apoptosis and undergo plasma cell differentiation. In this regard, the T follicular regulatory (T_FR_) cell subset (259) has yet to be described within TLO. The T_FR_ cell subset phenotypically resembles the T_FH_ cell subset in surface expression of CXCR5, PD-1, ICOS, and in positioning within the B cell follicle, but expresses the master transcription factors (FoxP3 and Bcl-6) for both the Treg and T_FH_ cell subsets (260–262). The precise role of the T_FR_ cell subset is still debated, with recent papers variably suggesting that T_FR_ cells are either specific (263) or not specific (264) for target antigen, but their relatively late ingress to the follicle suggests that they do not inhibit development of the GC, and that they either prevent escape of autoimmune variants that arise following SHM (260) or hasten termination of the response (265). This last role may be particularly pertinent to GC activity within TLO, which is characterized by its longevity, and which, consequently, may be more prone to subversion to autoreactivity; either because of failure of negative selection or because of external seeding with newly activated naïve or memory B cells (184, 203, 266, 267).

**Figure 2:**
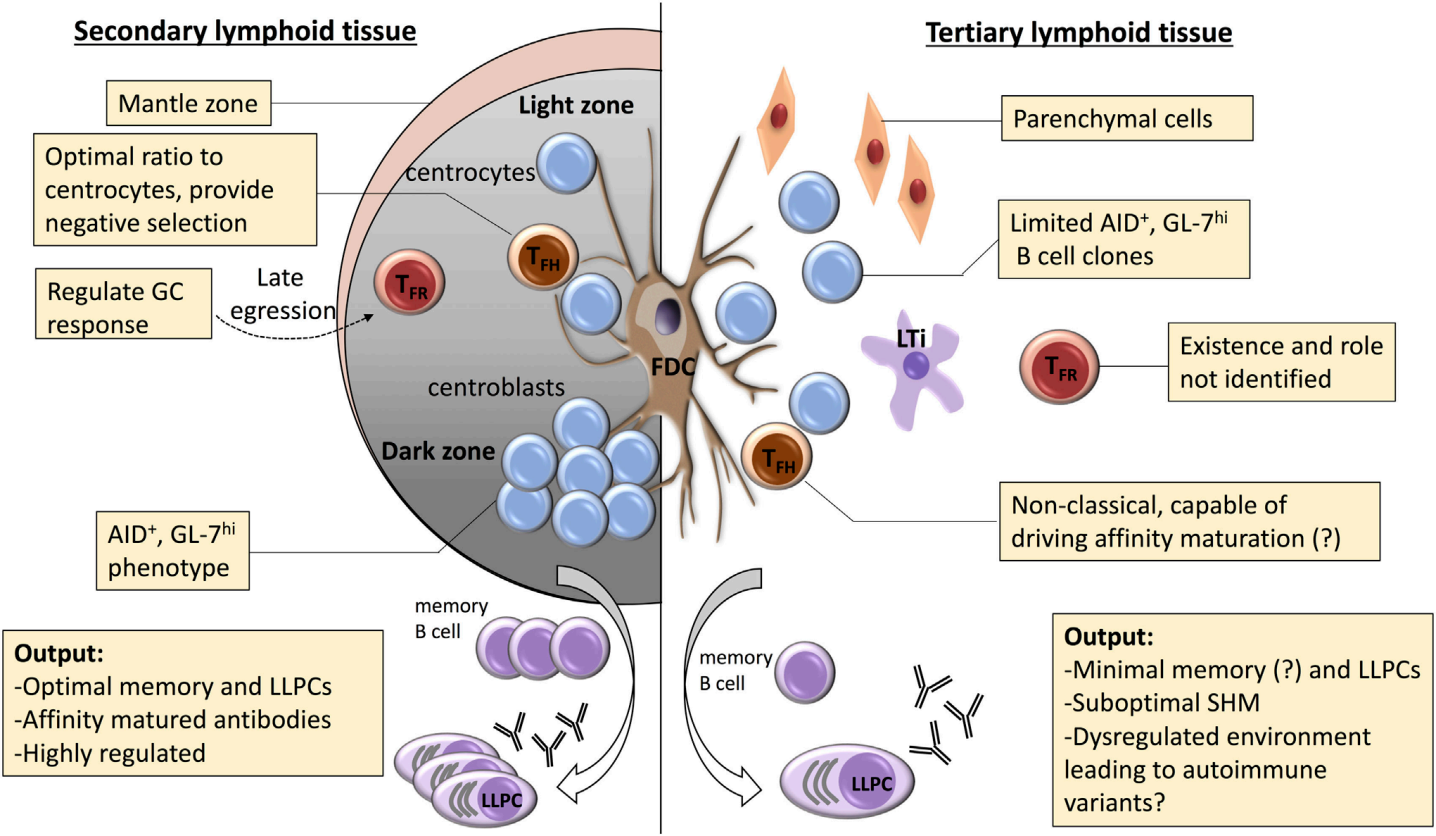
Potential mechanisms for dysregulated selection within tertiary lymphoid organs (TLOs). A number of mechanisms are responsible for regulating the germinal center (GC) response within conventional secondary lymphoid organs. Affinity maturation is critically dependent upon coordinated recycling through the dark zone, with competition for limiting number of T follicular helper (T_FH_) cells critical for selection of high-affinity clones. Effective selection is also dependent upon robust processes for destruction of low-affinity clones or those that have mutated to autoreactivity. These are less well understood, but include: optimization of T_FH_ cell numbers; negative input from T follicular regulatory (T_FR_) cells; and effective engulfment and disposal by tingible body macrophages. In TLOs, dark/light zone segregation is conspicuously absent, and the role of the T_FH_ cell remains poorly understood. Similarly, the T_FR_ cell population has yet to be characterized. Thus, although GC-type features are frequently described within TLO, it is likely that functional output of these GC-like structures differs from canonical secondary lymphoid function. We propose that the dysregulated nature of the GC response within TLOs favors the escape of autoreactive variants and developing of long-lasting humoral autoimmunity.

Aside for disorders in T_FH_ cell function, there are several other mechanisms by which the development of autoreactivity is possibly favored within a TLO (see Figure 2). A number of B cell intrinsic pathways have been identified that inhibit development of GC autoimmunity. These include expression of ELL-associated factor 2 (268), and signaling *via* TLR9 (269, 270) or inhibitory FcyRIIB ligand (271–273). Whether these signaling pathways are somehow modulated within a GC TLO to favor development of autoreactivity is, however, not known. B cell inhibition *via* FcyRIIB binding is dependent upon immune complexes simultaneously engaging the BCR, and thus local perturbations in effector antibody concentration, or differences in sialylation at the antibody Fc region (274), could conceivably alter the degree of FcyRIIB-mediated inhibition within the TLO. Alternatively, as well as a reservoir for target antigen, the FDC network expresses a variety of cytokines (such as Il-6 and BAFF) and chemokines (CXCL13) thought critical for effective GC function (275). Das et al. have recently reported that, in response to TLR7 signaling, the FDC can also promote autoimmunity, by secreting pro-inflammatory IFN-α (276). Thus, the inflammatory state thought responsible for triggering formation of a peripheral TLO may program an activated FDC phenotype that promotes subsequent diversification of the GC response to encompass autoreactive targets. Heightened cell turnover within the inflammatory milieu of the TLO may additionally increase the likelihood of autoimmunity developing, by overloading the capacity of tangible body macrophages to clear apoptotic bodies within the GC (277, 278).

## Conclusion

In summary, although components of the B cell GC response are frequently identified within TLOs, the precise function of these putative GCs, and how they compare to GCs within canonical SLOs, has yet to be determined. It seems likely from the expression of AID (32, 146, 147, 149, 151) and from clonal analysis of the constituent B cells (154), that SHM can occur. However, as discussed, we now know that physical segregation of the GC into dark and light zones is crucial for effective SHM, as this enables limiting numbers of T_FH_ cells within the light zone to inform the subsequent proliferative response of circling GC B cells in the dark zone. Such readily identifiable dark/light zone configuration is conspicuously absent from reported GCs within TLOs, and when allied to concerns relating to T_FH_ cell dysfunction, we propose that it is likely that affinity maturation within a TLO is at best sub-optimal, and certainly not as effective as within canonical lymphoid tissue. This is supported by the limited evidence available (157). Instead, it seems probable that dysregulated or uncoordinated responses within the TLO favor a breakdown of negative selection, with subsequent epitope diversification to encompass autoimmune variants. In this respect, we have previously reported, in a murine model of chronic heart graft rejection, that transplantation of MHC class II mismatched heart grafts triggers long-lasting anti-nuclear IgG autoantibody responses in the recipient (279) and is associated with development of intra-allograft TLO with prominent B cell features (32). Our recent work has highlighted that help for the development of humoral autoimmunity is provided by an unusual form of “peptide-degenerate” (but cognate) interaction between recipient autoreactive B cells and donor CD4 T cells that are passengers with the heart graft (280). Surprisingly, although triggered by donor CD4 T cell graft-versus-host recognition of MHC class II determinants on recipient B cells, maintenance of splenic and intra-allograft GC activity is dependent upon provision of help from T_FH_ differentiation of a recipient CD4 T cell subset. The chronic GC response that ensues is associated with spreading to encompass autoantibody responses against vimentin protein (281). This model, therefore, provides the opportunity, which our ongoing work will address, to clarify the relationship between aberrant CD4 T cell help and diversification of the TLO humoral autoimmune response to target new, previously quiescent epitopes which have been previously shown to be associated with detrimental graft function (282).

## Author Contributions

All authors contributed equally to the design and preparation of this review. JA configured the summary table and figures.

## Conflict of Interest Statement

The authors declare that the research was conducted in the absence of any commercial or financial relationships that could be construed as a potential conflict of interest.
